# Emerging treatment options for cryptosporidiosis

**DOI:** 10.1097/QCO.0000000000000761

**Published:** 2021-10-01

**Authors:** Melissa S. Love, Robert K.M. Choy

**Affiliations:** 1 Calibr, a division of The Scripps Research Institute, La Jolla, California, USA; 2 PATH, Seattle, Washington, USA

**Keywords:** *Cryptosporidium*, cryptosporidiosis, diarrhea, drug development, low-resource settings

## Abstract

**Purpose of review:**

Substantial progress has been made recently on the development of new therapeutics for cryptosporidiosis, an infection by the protozoan parasite *Cryptosporidium* that is associated with diarrhea, malnutrition, growth stunting, cognitive deficits, and oral vaccine failure in children living in low-resource settings.

**Recent findings:**

Various drug discovery approaches have generated promising lead candidates. The repurposed anti-mycobacterial drug clofazimine was tested in Malawian HIV patients with cryptosporidiosis but was ineffective. Target-based screens identified inhibitors of lysyl-tRNA synthetase, phenylalanyl-tRNA synthetase, methionyl-tRNA synthetase, and calcium-dependent protein kinase 1. Phenotypic screens led to discovery of a phosphatidylinositol 4-kinase inhibitor, the piperazine MMV665917, and the benzoxaborole AN7973. The relationship between pharmacokinetic properties and *in vivo* efficacy is gradually emerging. A pathway to clinical trials, regulatory approval, and introduction has been proposed, but additional work is needed to strengthen the route.

**Summary:**

Several lead compounds with potent activity in animal models and a favorable safety profile have been identified. A sustained effort will be required to advance at least one to clinical proof-of-concept studies. The demonstrated risk of resistance indicates multiple candidates should be advanced as potential components of a combination therapy.

## Introduction

Infection by the protozoan intestinal parasite *Cryptosporidium* spp. is a leading cause of diarrheal disease morbidity and mortality [1, 2]. Recent estimates suggest *Cryptosporidium* is responsible for approximately 7.6 million cases and between 48,000 – 202,000 deaths annually among young children in low-resource settings [3, 4]. Beyond acute morbidity and mortality, both symptomatic and asymptomatic infections are associated with long-term sequelae including growth stunting, malnutrition, and cognitive development deficits [5, 6]. Sporadic outbreaks from contaminated water supplies are common even in high-income countries [7]. *C*. *hominis* and anthroponotic *C*. *parvum* are most commonly identified in humans; person-to-person is the predominant mode of transmission [8], although zoonotic species are occasionally found in humans.

Despite the substantial disease burden caused by *Cryptosporidium,* treatment options remain limited. The only treatment approved by a stringent regulatory authority (the US Food and Drug Administration) is nitazoxanide, a drug that is efficacious in otherwise healthy adults, but has marginal efficacy in malnourished children and is no better than placebo in immunocompromised HIV-positive patients [9]. Another limitation is that nitazoxanide is not approved for children younger than 12 months, the most vulnerable patient population. In the absence of highly effective specific therapy and a simple, point-of-care diagnostic test [10], in most low-resource settings cryptosporidiosis is treated symptomatically with either oral or intravenous rehydration. A target product profile and use-cases of an ideal anti-cryptosporidial therapeutic have been proposed [11, 12].

**Figure 1:**
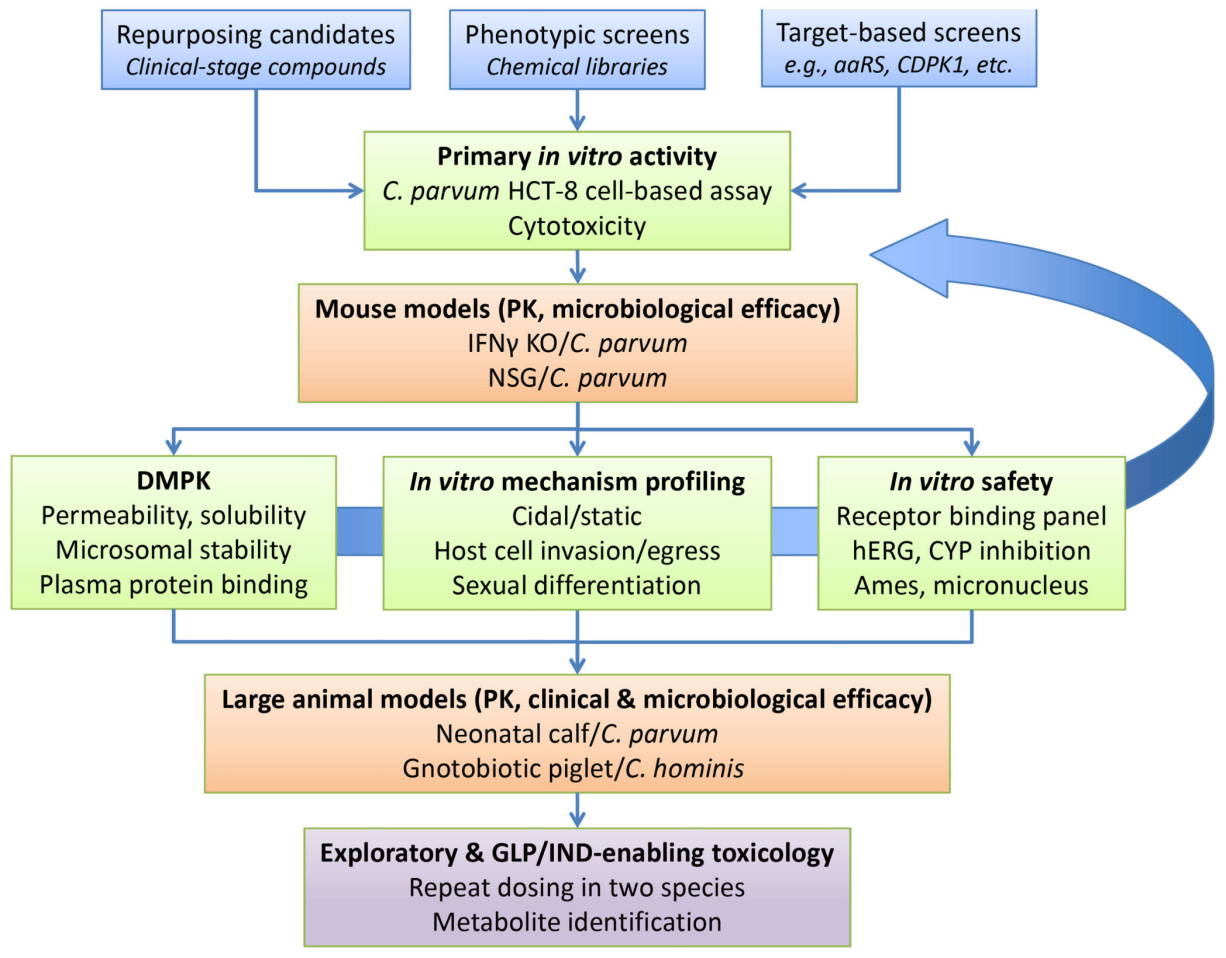
Screening cascade for cryptosporidiosis therapeutic candidates. Starting compounds can be identified from collections of clinical-stage compounds, phenotypic screening of chemical libraries, or screens of previously characterized or rationally-selected targets. Regardless of the source, compounds are first screened for activity in a standardized cell-based assay such as *C*. *parvum* infection of HCT-8 cells. Hits are counterscreened for cytotoxic effects and typically a 20-fold or higher margin is required for progression. The next step is characterizing both PK and microbiological efficacy as determined by reduction in oocyst shedding in a mouse model. Compounds with *in vivo* activity are then characterized for several parameters in parallel, including DMPK properties, *in vitro* safety assays, and further *in vitro* profiling, such as for activity at specific stages of the parasite lifecycle. This may involve an iterative process of repeated medicinal chemistry optimization and retesting with *in vitro* and *in vivo* assays (curved arrow). Only compounds with confirmed drug-like properties are tested in expensive and limited-capacity large animal models for PK and clinical (reduction in diarrheal stool output) and microbiological efficacy. Only compounds with demonstrated large animal efficacy would be advanced to exploratory (non-GLP) toxicology studies to de-risk and then GLP toxicology to enable IND-filing and clinical testing. *Abbreviations*: aaRS, aminoacyl-tRNA synthetase; CDPK1, calcium dependent protein kinase 1; CYP, cytochrome P450; DMPK, drug metabolism and pharmacokinetics; hERG, human Ether-a-go-go-Related Gene; GLP, Good Laboratory Practices; IFNγ KO, interferon gamma knockout; IND, Investigational New Drug application; NSG, NOD SCID gamma; PK, pharmacokinetics.

Since *Cryptosporidium* emerged as a major contributor to the global diarrheal disease burden in the past decade, a number of academic and industrial groups have advanced promising new chemical entities (NCEs) through a rigorous screening cascade (Figure 1). The past two years have seen substantial progress with development of many NCEs for cryptosporidiosis, thus the purpose of this review is to provide an overview of these projects.

## Pipeline of emerging cryptosporidiosis therapeutics

In this review, we discuss recent advancements around seven candidates in development as cryptosporidiosis-specific therapeutics. These compounds emerged from either a target-based approach of a known or validated target in other pathogens, or a phenotypic whole-cell screen against *Cryptosporidium* parasites with a curated compound library (Table 1).

### Candidates from target-based screens

While target-based therapeutic screening has historically been perceived to be challenging in *Cryptosporidium* because it appropriates many essential metabolites from host cells, a number of promising candidates have emerged from target-based screens. One class is inhibitors of aminoacyl-tRNA synthetases (aaRSs) – enzymes that charge amino acids for polypeptide synthesis and thus tend to be essential for growth. Three will be considered in detail below, along with calcium-dependent protein kinase 1 (CDPK1).

#### Lysyl-tRNA synthetase (KRS)

KRS was originally identified as the target of cladosporin in *P*. *falciparum.* Based on structural homology, *P*. *falciparum* KRS (PfKRS) inhibitors were tested on *C*. *parvum* KRS (CpKRS) and a consortium of investigators led by University of Dundee recently published a comprehensive study of early lead compounds (subsequently referred to in this review as “leads”) targeting both enzymes [13]. A potent chromone compound was reported with excellent drug-like properties (Table 1). Because of toxicity observed in rodents, this early lead was deprioritized; however, it provided important validation of KRS as a drug target. Subsequent chemical refinement has identified further optimized leads with greater potency and less toxicity that will be tested in the neonatal calf efficacy model (Baragaña B, personal communication). The early lead chromone has high solubility and oral bioavailability (Table 1), which likely contribute to its high systemic exposure. However, a later lead compound retained efficacy in mouse models with relatively lower systemic exposure. The basis for this disconnect is under investigation. Molecular modeling comparing *Pf*KRS and *Cp*KRS with human KRS suggested a potential mechanism for selectivity, which would reduce risks of on-target toxicity.

#### Phenylalanyl-tRNA synthetase (PheRS)

Like KRS, PheRS was also initially validated as a drug target for *P*. *falciparum* and then subsequently explored as a target for *Cryptosporidium* by a consortium of academic and industrial collaborators. In an effort led by the Broad Institute, a curated set of compounds with previously established antimicrobial activity and known mechanisms of action (MoA) was screened, and a bicyclic azetidine with potent activity against *C*. *parvum in vitro* (Table 1) was identified [14]. Homology with PfPheRS was beneficial in rapidly establishing a structure-activity relationship for CpPheRS inhibitors and identifying a range compounds with varying pharmacokinetic (PK) and physiochemical properties, including differences in oral bioavailability, volume of distribution, metabolic half-life, and solubility. Several compounds with a range of properties were then tested *in vivo* using the NOD SCID gamma (NSG) mouse model and a direct relationship between higher bioavailability and greater *in vivo* activity was demonstrated. As a confirmation of the MoA of bicyclic azetidines on PheRS, CRISPR-Cas9 was used to introduce a resistance-conferring mutation found in PfPheRS into CpPheRS, which afforded resistance to BRD7929, a representative compound.

#### Methionyl-tRNA synthetase (MetRS)

Exploration of MetRS inhibitors for cryptosporidiosis arose from a program at the University of Washington investigating this enzyme as a target for other pathogens including *Trypanosoma brucei.* Crystal structures of the TbMetRS bound to inhibitors were leveraged to identify the homologous binding sites in *C*. *parvum* and *C*. *hominis* MetRS, which retain 76% identity to *T. brucei.* Several TbMetRS inhibitors were shown to potently inhibit CpMetRS *in vitro* and suppress oocyst shedding in both the IFNγ KO and NSG mouse models [15]. Pharmacokinetic analysis of CpMetRS inhibitors found the compounds with highest activity in mouse models had both high systemic exposure (Table 1) and high levels in feces (>10 μM), therefore it was not possible to discern whether systemic or intestinal luminal exposure is more important for activity with this compound class. In the neonatal calf efficacy model [16], lead compound 2093 initially strongly suppressed oocyst shedding and diarrheal symptoms within the first four days post-infection, but then diarrhea and oocyst shedding rebounded in 2 of 3 animals. Subsequent sequencing of fecal samples revealed the acquisition of either a D243E or T246I mutation in CpMetRS. Structural modeling indicated these mutations disrupted compound binding to CpMetRS. *In vitro* studies with recombinant enzymes containing these mutations were >170-fold less sensitive to inhibition by compound 2093, and *C*. *parvum* parasites engineered with either mutation via CRISPR-Cas9 were found to be 613-fold (D243E) or 128-fold (T246I) less sensitive to compound 2093. These results demonstrate that resistance to *Cp*MetRS inhibition arose rapidly *in vivo* and necessitate caution for future development and introduction of cryptosporidiosis therapeutics.

#### Calcium-dependent protein kinase 1 (CDPK1)

CDPKs are an attractive drug target for apicomplexan diseases because they are essential and have no analogous proteins in mammals [17]. Bumped kinase inhibitors (BKIs) are ATP-competitive inhibitors of CDPKs and named for a structural bump that prevents binding in kinases with larger “gatekeeper” residues in the ATP-binding pocket [18]. Researchers at the University of Washington discovered that a selection of *Toxoplasma gondii* CDPK1 (*Tg*CDPK1) BKI compounds also inhibit *Cp*CDPK1 [19]. Medicinal chemistry efforts over the past decade have resulted in several hundred BKI compounds displaying a range of PK/PD properties and anti-parasitic activity [20], with the majority represented by three scaffolds: pyrrolopyrimidine (PrP), pyrazolopyrimidine (PP), and 5-aminopyrazole-4-carboximide (AC). Early leads showed good efficacy in mouse, calf, and gnotobiotic (GB) piglet models of cryptosporidiosis, though this series of compounds (subsequently referred to in this review as “series”) has faced challenges related to cardiovascular toxicity, teratogenicity, and varying efficacy due to the differing PK/PD parameters across scaffolds [21, 22].

Due to ongoing toxicity liabilities in the PP and PrP scaffolds [23], the most promising candidates have come from the AC scaffold. A recent report identified two AC-scaffold preclinical leads (BKI-1708 and BKI-1770) with good *in vitro* potency against both the *Cp*CDPK1 enzyme and cells, minimal human Ether-a-go-go Related Gene (hERG) activity, and good efficacy and safety in a mouse model of cryptosporidiosis (Table 1) [24]. Within the AC scaffold series, high solubility but not high plasma or fecal exposure correlated with *in vivo* efficacy. Both BKI-1708 and BKI-1770 show good *in vivo* efficacy and alleviate many of the toxicity and safety liabilities of the BKI series, and warrant further study as preclinical candidates.

### Candidates from Phenotypic Screening Efforts

Another approach for finding new cryptosporidiosis therapeutics is phenotypic screening. Phenotypic screens are less biased and not dependent on a known target, and several emerging therapeutics for cryptosporidiosis were discovered from screening compound collections with known activity in other pathogens against *Cryptosporidium* spp. [25]. Phenotypic screens may identify compounds with novel targets or pathways, with the caveat that target identification and MoA studies are often needed after active compounds are discovered. There have been a number of additional phenotypic screens for *Cryptosporidium* drug discovery [25, 26], though the compounds discussed in this review are the most advanced.

#### Phosphatidylinositol 4-kinase 4 [PI(4)K] inhibitors

Pyrazolopyridine inhibitors of PI(4)K (a validated malaria drug target) were discovered from a phenotypic screen of 6,220 parasite actives. Lead compound KDU731 was shown to have potent *in vitro* activity against *C*. *parvum* and *C. hominis,* excellent efficacy in mouse and calf models of cryptosporidiosis (Table 1) and demonstrated safety in various *in vitro* tests and a rat toxicology study [27]. Further *in vitro* activity profiling showed that a related analog, KDU691, was parasiticidal at its EC_90_, and its MoA was impediment of merozoite formation [28], likely due to impairment of lipid membrane processing from inhibition of CpPI(4)K. Interestingly, PK of KDU731 in *C.* parvum-infected calves showed limited systemic exposure, indicating that it may not be required for clinical efficacy in this series.

#### MMV665917

A phenotypic high-content imaging screen of the Medicines for Malaria Venture (MMV) Malaria Box against *C*. *parvum* conducted by researchers at the University of Vermont discovered MMV665917, a novel piperazine-based compound that is specific for *Cryptosporidium* parasites and blood-stage *Plasmodium* spp. [29]. While the molecular target is unknown, *in vitro* time of action assays indicate it may affect the transition between asexual and sexual life stages [28, 30]. Excitingly, this molecule shows efficacy in both the IFNγ KO and NSG mouse models of cryptosporidiosis, as well as in the GB piglet and dairy calf models [31, 32]. MMV665917 PK studies in uninfected neonatal calves showed high exposures in both the serum and feces (Table 1), though it is unclear if exposure in both compartments is necessary for *in vivo* efficacy. The compound was well tolerated in animals, and no significant organ toxicity was observed; however, MMV665917 partially inhibits hERG, indicating a potential for cardiac toxicity. It is possible that medicinal chemistry efforts can reduce the hERG inhibition potential of this scaffold while retaining the excellent anti-cryptosporidial activity, and the identification of the molecular target could greatly aid in these efforts.

#### AN7973

Another molecule that has emerged from a phenotypic screen of a focused collection of malaria active compounds is the benzoxaborole AN7973. Similar to MMV665917, AN7973 does not have a confirmed target or MoA but it has potent *in vitro* activity against both *C*. *parvum* and *C. hominis,* and shows efficacy in both the IFNγ KO and NSG mouse models as well as the calf model of cryptosporidiosis [28, 33]. In PK studies of AN7973 in mice and dairy calves, the compound displayed high plasma exposure, a long half-life (Table 1), high fecal exposure, and was well-tolerated in rodents and calves. The putative target is Cleavage and Polyadenylation Specific Factor 3 (CPSF3) based on the inhibition of PfCPSF3 and TgCPSF3 by related benzoxaborole compounds and the shared catalytic core homology of this enzyme between these apicomplexan parasites [34], though more work is needed to confirm this as the target of AN7973 and related compounds in *Cryptosporidium.*

### PK/PD drivers of *in vivo* efficacy

To understand the ideal PK profile for an *anti-Cryptosporidium* drug, researchers have explored various approaches including empirical observations of systemic and intestinal exposure, physiologically-based PK modeling [35], and consideration of efflux pumps [36]. While a broadly applicable profile remains elusive, in some cases such as the BKIs and PI(4)K inhibitors, compound concentrations in intestinal epithelial cells seem to drive efficacy. In contrast, with PheRS inhibitors systemic concentrations are most closely correlated with efficacy. Further work is needed to determine whether more broad observations can be made, or whether drivers are specific to individual targets, MoAs, or chemical series. Having clearly defined physiochemical and PK properties that drive *in vivo* and clinical efficacy for cryptosporidiosis therapeutics would help to prioritize emerging scaffolds and allow for efficient allocation of resources within drug discovery programs.

## Clinical trial of clofazimine and lessons for future proof-of-concept studies

A repurposing candidate that emerged from a phenotypic screen, clofazimine (CFZ) [37], was quickly advanced into a clinical trial to assess its safety and efficacy in HIV-infected patients with cryptosporidiosis [38]. This Phase 2a randomized, double-blind, placebo-controlled study had two parts: Part A had primary outcomes of safety, PK, and reduction in *Cryptosporidium* oocyst shedding; Part B was an open-labeled study comparing the PK of matched HIV-positive individuals without cryptosporidiosis (or diarrhea). The study faced many unexpected challenges with study initiation, population, implementation, and cultural issues [39], though the sponsors were able to find solutions to successfully reach the endpoint. The study found treatment with CFZ had no significant impact on *Cryptosporidium* oocyst shedding, diarrheal episodes, stool weight, or consistency scores. Of note, the Part A participants (with diarrhea) had about 2-fold less plasma exposure of CFZ as compared to Part B participants (no diarrhea), though no conclusions could be made whether this impacted the lack of efficacy. While the results of the study do not support the efficacy of CFZ for treatment of cryptosporidiosis in a severely immunocompromised HIV population, this trial served to lay essential groundwork for future human studies to assess the efficacy of potential new anti-cryptosporidials [40].

In the past decade, increased awareness and funding toward finding a *Cryptosporidium-specific* therapeutic has resulted in a reasonably diverse pipeline; however, there is still a lack of a clinical proof of concept for a drug that is equivalent or superior to nitazoxanide. Specific use-case scenarios may also dictate the best clinical path for emerging therapeutics for cryptosporidiosis [12]. Other practical considerations of treatment settings include the need for simple and affordable point-of-care diagnostics as well as reliable access to cryptosporidiosis treatments, i.e., wherever children with diarrhea receive medical care and at nutritional rehabilitation and HIV treatment centers. These requirements are the most difficult to achieve in low-resource settings where the burden of *Cryptosporidium* infection is the highest. Accessible diagnostics and clearly defined diagnosis criteria are essential for deciding how to treat patients with new anti-cryptosporidials [41], especially because there is a lack of guidance around empiric treatment or mass drug administration campaigns.

Another possible solution for deconvoluting the clinical path for emerging treatments for cryptosporidiosis could be a controlled human infection model (CHIM). It is feasible that the clinical pathway for anti-cryptosporidial NCEs may diverge between the different target populations and use cases, and therefore these studies could serve as a small-scale first pass to determine proof-of-concept efficacy of NCEs before a large financial investment in specific studies involving immunocompromised patients or young children. Although there is interest and precedence of CHIM for cryptosporidiosis [42], the most recent studies were conducted nearly two decades ago, and new regulatory requirements present unique challenges that complicate the re-establishment of this model [43].

To further complicate matters, the spontaneous resistance to CpMetRS inhibitor 2093 in the neonatal calf efficacy model [16] is extremely alarming and necessitates thoughtful design of future clinical trials and eventual implementation of new treatments. More studies are needed, ideally in early stages of development, to determine the frequency at which resistance mutations arise for this drug and others in the pipeline. Combination therapies and regimens are essential in combating other diseases with a large global health burden (e.g., malaria, HIV, tuberculosis), and the rollout of an effective therapy for cryptosporidiosis may quickly be rendered useless or exacerbate the burden of *Cryptosporidium* infection. Of note, there are currently no studies looking at any emerging therapeutics in combination.

## Conclusions

The field of drug discovery for *Cryptosporidium* has made great strides in a short amount of time through both targeted approaches and phenotypic high-throughput screens; however, there is likely to be attrition of compounds as they progress through the later stages of development. The evolution of the clinical path for new anti-cryptosporidial compounds must allow for advancement of promising compounds for key target populations and use-case scenarios while balancing the risks that may push away support from the pharmaceutical industry. The CRYPTOFAZ study was invaluable for lessons learned and capacity building efforts for future trials in resource-limited settings. Finally, implementation strategies of new drugs are critical. Even the best drug for treating *Cryptosporidium* infection will have limited impact if it cannot be effectively implemented with accessible and accurate diagnostics, and monotherapy strategies may be problematic if spontaneous resistance selection is observed with other compounds.

## Figures and Tables

**Table:** 

Lead Compound	Development Phase	Target	*in vitro* Activity			*in vivo* Efficacy					Pharmacokinetic Profile						
			*C*. *parvum* (Iowa) EC_50_ (μM)	*C*. *hominis* (TU502) EC_50_ (μM)	Enzymatic IC_50_ (μM)	IFNγ KO mouse	NSG mouse	Dairy Calf	GB Piglet	Human	Dose	C_max_	AUC_0-last_	F%	T_1/2_	V_dss_	Comments
Nitazoxanide	Launched	*Cp* PFOR; possible modulation of host interferons	2.84 ± 1.90	2.82 ± 0.45	NA	No	No	No	Efficacious at 100 mg BID for 10 days	Yes in immunocompetent individuals	100 mg single dose (oral suspension, children 1–3 years)	3.11 μg/mL	11.7 μg·hr/L	NR	1.03–1.6 hr	NR	All reported parameters are based on the metabolite, tizoxanide; tizoxanide is highly protein bound (>99%)
Clofazimine	Clinical (Phase IIa)	Unknown	0.0149 ± 0.0085	0.341 ± 0.302	NA	Efficacious at 10 mg/kg QD	No	Weakly active at 30 mg/kg (unpublished)	ND	No	100 mg Lamprene (Day 5; Part A and B in clinical trial)	280.7 ng/mL (Part A); 514.1 ng/mL (Part B)	6,863 ng·hr/mL (Part A); 11,298 ng·hr/mL (Part B)	NR	336.5 hr (Part A); 535.5 hr (Part B)	NR	Low oral bioavailability; high lipophilicity and permeability in GI tract; Clinical trial data showed about 2-fold less plasma exposure in participants without diarrhea (Part B); Less than 2% of the cumulative CFZ doses was recovered in stool in both groups over the five days of stool collection
KDU731; *Cp*PI4K-SD Lead	Preclinical	*Cp* PI(4)K	0.063 ± 0.028	0.130 ± 0.074	0.025 ± 0.004	Efficacious at 10 mg/kg QD	ND	Efficacious at 5 mg/kg BID	ND	ND	KDU731: 2.3 mg/kg (oral, mouse); 5 mg/kg (IV, mouse); 5 mg/kg (oral, calf)	406 nM (oral, mouse); 228 nM (calf)	2306 nM·hr (oral, mouse); 1909 nM·hr (calf)	37% (oral, mouse)	2.47 hr (oral, mouse)	1.12 L/kg (IV, mouse)	No correlation between efficacy and plasma exposure
BKI-1708	Preclinical	*Cp* CDPK1	0.41	NR	0.0007	Efficacious at 8 mg/kg BID (BKI-1708); many others	Efficacious at 10 mg/kg BID (BKI-1553); others	Efficacious at 5 mg/kg BID (BKI-1369); others	Efficacious at 10 mg/kg BID (BKI-1369)	ND	BKI-1708: 10 mg/kg (oral, mouse)	2.9 μM	247.1 μmol·min/L	NR	42.6 min	NR	Varied PK/PD across 3 series scaffolds; GI exposure necessary for efficacy; no correlation with plasma exposure
AN7973	Discovery (Late lead)	*Cp* CPSF3 (putative)	0.13–0.43	0.63	NA	Efficacious at 10 mg/kg QD	Efficacious at 10 mg/kg QD	Efficacious at 10 mg/kg QD	ND	ND	10 mg/kg (oral, mouse); 5 mg/kg (oral, calf)	8.63 μg/mL (mouse); 3.57 μg/mL (calf)	92.7 μg·hr/mL (mouse); 190 μg·hr/mL (calf)	37% (mouse)	6.6 hr (mouse); 31 hr (calf)	NR	Half life ~5x greater in calves than mice; high concentrations found in feces
Compound 2093	Discovery (Late lead)	*Cp* MetRS	0.006–0.029	0.015	0.0009 ± 0.0004	Efficacious at 25 mg/kg BID	Efficacious at 50 mg/kg BID	Initial efficacy, then resistance observed in calf model at 15 mg/kg BID	ND	ND	50 mg/kg (oral, mouse)	5.8 μM	1863 μmol·min/L	NR	NR	NR	Calf PK and efficacy studies: plasma and fecal levels >3x EC90 for over 24h
MMV665917	Discovery (Late lead)	Unknown	1.9–2.3	4.1	NA	Efficacious at 30 mg/kg BID	Efficacious at 30 mg/kg BID	Efficacious at 22 mg/kg QD	Efficacious at 20 mg/kg BID	ND	55 mg/kg (oral, mouse); 22 mg/kg (oral, calf); 10 and 20 mg/kg (oral, piglet)	NR	NR	NR	NR	NR	PK in healthy mice: high fecal and plasma concentrations with sustained exposure; PK from infected calf model: sustained fecal and serum concentrations >3x EC90; PK from infected piglet model: plasma exposure remained >3x EC90; gut contents showed concentrations > EC90 60h after treatment ended
Compound 5	Discovery (Late lead)	*Cp* KRS	1.3	6.0	0.13	Efficacious at 20 mg/kg QD	Efficacious at 20 mg/kg QD	ND	ND	ND	10 mg/kg (oral, mouse); 3 mg/kg (IV, mouse)	5.4 μg/mL (oral)	1,300–3,000 μg·min/mL (oral)	100%	2.5 hr (IV)	1 L/kg (IV)	Very high oral bioavailability
BRD7929	Discovery (Lead Op)	*Cp* PheRS	0.008–0.073	0.010	0.060 (*Ch* PheRS)	ND	Efficacious at 10 mg/kg QD	ND	ND	ND	1 mg/kg (oral, mouse); 0.6 mg/kg (IV, mouse)	NR	NR	80%	32 hr (IV)	29 L/kg	BRD7929 has high oral bioavailability, volume of distribution, and solubility; compounds in series with higher bioavailability had better efficacy, possibly due to permeability

NA = Not Applicable ND = Not Determined NR = Not Reported EC_50_ = Half-maximal effective concentration IC_50_ = Half-maximal inhibitory concentration GB Piglet = Gnotobiotic piglet model (C. hominis) C_max_ = maximum or peak serum (plasma) concentration of a drug after a single dose AUC_0–last_ = Area under the curve; time-averaged concentration of drug in plasma F% = Oral bioavailability T_1/2_ = Half-life in plasma V_dss_ = Volume of distribution at steady state

## Key points

- Cryptosporidiosis is a serious parasitic infection of young children in low-resource settings with insufficient treatment options.
- In recent years, several promising small molecule therapeutic candidates have advanced through discovery and a repurposed compound has been tested in a Phase 2 study in Malawi.
- More work is needed to define a clear pathway along which to advance additional candidates to clinical proof-of-concept.
- Risk of resistance to monotherapies necessitates careful consideration of future clinical study design and plans for implementation of effective therapeutics.
